# Arpeggio: A Web Server for Calculating and Visualising Interatomic Interactions in Protein Structures

**DOI:** 10.1016/j.jmb.2016.12.004

**Published:** 2017-02-03

**Authors:** Harry C Jubb, Alicia P Higueruelo, Bernardo Ochoa-Montaño, Will R Pitt, David B Ascher, Tom L Blundell

**Affiliations:** 1 Department of Biochemistry, Sanger Building, University of Cambridge, 80 Tennis Court Road, Cambridge CB2 1GA, UK; 2 UCB, 208 Bath Road, Slough, West Berkshire SL1 3WE, UK

**Keywords:** PDB, Protein Data Bank, SIFt, structural interaction fingerprint, protein interactions, protein–protein interactions, protein–ligand interactions, molecular recognition, drug design

## Abstract

Interactions between proteins and their ligands, such as small molecules, other proteins, and DNA, depend on specific interatomic interactions that can be classified on the basis of atom type and distance and angle constraints. Visualisation of these interactions provides insights into the nature of molecular recognition events and has practical uses in guiding drug design and understanding the structural and functional impacts of mutations. We present Arpeggio, a web server for calculating interactions within and between proteins and protein, DNA, or small-molecule ligands, including van der Waals', ionic, carbonyl, metal, hydrophobic, and halogen bond contacts, and hydrogen bonds and specific atom–aromatic ring (cation–π, donor–π, halogen–π, and carbon–π) and aromatic ring–aromatic ring (π–π) interactions, within user-submitted macromolecule structures. PyMOL session files can be downloaded, allowing high-quality publication images of the interactions to be generated. Arpeggio is implemented in Python and available as a user-friendly web interface at http://structure.bioc.cam.ac.uk/arpeggio/ and as a downloadable package at https://bitbucket.org/harryjubb/arpeggio.

## Highlights

• Enumeration and visualisation of molecular interactions can facilitate drug development and provide insights towards understanding the consequences of mutations in genetic diseases and protein engineering.
• Reliable and comprehensive methods to evaluate and visualise the full range of potential molecular interactions across many atom types present in protein structures are invaluable.
• Arpeggio calculates all intra- and interatomic interactions in macromolecular structures, including van der Waals', ionic, carbonyl, metal, hydrophobic, and halogen bond contacts, and hydrogen bonds and specific atom–aromatic ring (cation–π, donor–π, halogen–π, and carbon–π) and aromatic ring–aromatic ring (π–π) interactions, within a provided Protein Data Bank file. Calculations can be within or between any combination of protein, DNA, or small organic molecules.
• The Arpeggio web server (http://bleoberis.bioc.cam.ac.uk/arpeggioweb/) was implemented to provide a freely available, user-friendly web interface for the exploration of molecular interactions within protein structures, including through WebGL-based visualisation of interactions and downloadable interactive PyMOL session files.
• Arpeggio is written in Python, requires only Open Source dependencies, and is freely available for download at https://bitbucket.org/harryjubb/arpeggio for use in custom analyses.

## Graphical Abstract

**Figure:**
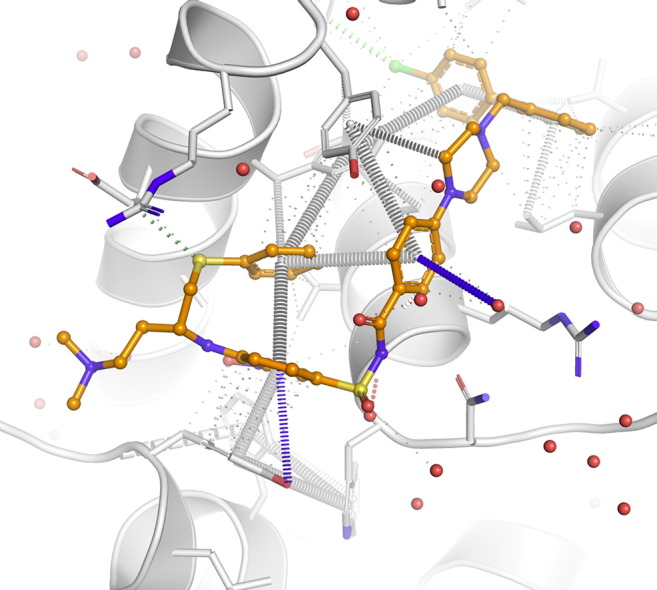

### Introduction

Molecular recognition is driven in part by the favourable matching of chemistry between two or more molecules. Many known interactions in molecular recognition can be represented by pairwise contacts between atoms [1], [2], [3], [4], [5], [6], [7], [8]. While interatomic, non-bonded interactions, such as hydrogen bonding and π-stacking interactions, are generally intuitively recognised by the trained observer, it is helpful to visualise them based on defined criteria. Definition and enumeration/visualisation of interactions as opposed to intuition help to ensure that we have a more rigorous, impartial, and complete understanding of the nature of molecular binding sites. This allows the systematic evaluation of the interactions made in, for example, protein–ligand interactions, thus ensuring that key interactions are not overlooked [9]. Some tools are available that aid in this understanding, such as the Ligand Protein Contacts server [10] and GIANT [9], and specific programs for calculating individual interaction types such as polar contacts in PyMOL and hydrogen bonds with Joy [11], REDUCE [12], HBPLUS [5], Bioptools [13], and LIGPLOT + [14]. However, these tools use a limited set of interaction types and are confined to protein–ligand interactions for visualisation. FingerPrintLib [15] and PyPLIF [16] calculate multiple interaction types but are restricted to protein interactions with small organic molecule ligands only. PLIP [17] recently expanded these interactions to look at all protein–ligand interactions, but like earlier methods is limited to binary interaction fingerprints in its output.

We have previously published databases of calculated interatomic interactions covering the Protein Data Bank (PDB [18]) [19], [20], [21], [22], [23]. We now present Arpeggio, a web server for calculating interatomic interactions of 15 subtypes based on atom type, distance and angle terms. Arpeggio can be applied not only to protein–ligand interactions but also to protein–protein, protein–nucleic acid, and nucleic acid–nucleic acid interactions. The server can accept user-submitted structures in addition to PDB accession codes and thus can be used to calculate interactions for non-PDB structures such as homology models or docking poses. The web server provides downloadable tabular data enumerating interactions between molecular entities of interest for further analysis, in addition to WebGL- and PyMOL session-based visualisation of all interactions present in an input structure. The Arpeggio Python program that calculates interactions is Open Source (available at https://bitbucket.org/harryjubb/arpeggio), has only Open Source dependencies, and can be installed and run on Linux and Mac OSX.

### Results

#### Arpeggio program implementation

Arpeggio is implemented in Python and uses BioPython [24] and OpenBabel [25] to process PDB structure files. OpenBabel is used to assign atom types to each atom in the structure via SMARTS (a molecular pattern-matching language) queries, and BioPython's KDTree implementation is used to extract nearest-neighbour atoms within a 5-Å radial cutoff. Each pairwise interatomic contact is given a structural interaction fingerprint (SIFt) [26] using an expanded definition of the [15] interaction types. The first five bits of this fingerprint are mutually exclusive, and denote whether the interaction is a steric clash, covalent bond, van der Waals’ clashing (overlapping van der Waals’ radii, which can be common in structural models derived from X-ray crystallography), van der Waals'’ or "proximal". The first four bits are set based on theoretical covalent and van der Waals' radii defined in OpenBabel; other interactions that are still within the 5-Å cutoff are “proximal” but may not represent a “meaningful” interaction. The remaining bits correspond to specific “feature” interactions: hydrogen bonds, weak hydrogen bonds, halogen bonds, and ionic, metal complex, aromatic, hydrophobe–hydrophobe, and carbonyl interactions. We also added “polar” and “weak polar” contact types, which correspond to hydrogen bond and weak hydrogen bond interactions without angle terms; these are less sensitive to hydrogen placement.

Overall, the SIFt typifies the interactions made between a given atom pair and can be enumerated for groups of atoms, for example, a small-molecule ligand. Aromatic rings are also perceived using OpenBabel, and aromatic–aromatic ring (π–π) and atom ring (cation–π, donor–π, halogen–π, and carbon–π) interactions are recorded.

Atomic-resolution SIFts are enumerated for residues and are stored as binary and integer (counts of atoms making given contacts) fingerprints per residue. Aromatic–aromatic ring (π–π) and atom ring (cation–π, donor–π, halogen–π, and carbon–π) interactions are stored in the residue-level SIFts.

#### Improvements to definitions of interatomic contacts

Arpeggio builds on the SMARTS-based atom-typing and distance/angle-based contact definitions [19], [23]. We reviewed the SMARTS-based atom typing in CREDO and improved upon them. In some cases, we have modified the definitions themselves; for example, in the SMARTS queries for hydrogen bond acceptors, where the original CREDO SMARTS ruled out acceptors for carboxyl groups, we replaced these definitions with more lenient terms, allowing any covalently bound oxygen atom to be an acceptor. The SMARTS-based atom-typing definitions used in Arpeggio and built on from CREDO are available in the Arpeggio program configuration files, distributed with the source code, and are presented in Supplementary Data.

In other cases, CREDO's atom-typing SMARTS were sufficient; however, errors in protein structures due to poor resolution can cause incorrect atom typing. For example, where protomer termini do not have all atoms of the amino or carboxyl groups modelled due to comparatively flexible termini, atoms can be mislabelled. For example, a terminal carboxyl group missing an oxygen atom could be identified as a hydroxyl, and therefore, the lone oxygen atom would be typed as being a hydrogen bond donor in spite of not being covalently bound to a hydrogen atom. For protein termini and other polypeptide residues, this limitation was fixable by identifying the terminal residues of polypeptides using BioPython and by assigning the main-chain atom types from a dictionary of protein atom types, including distinction of hydroxyls and carboxyls from protein atom names. Combined with SMARTS-based atom typing, this feature gives Arpeggio a key advantage because of its ability to type atoms where protein atoms are missing and for modified residues and small-molecule ligands. In the Arpeggio program, a command-line option can be set to enable these definitions to be lenient with respect to tautomerism or other ambiguities, for example, the flipping of histidines.

#### Additional interatomic interaction types in Arpeggio

We added interaction types to Arpeggio that included group interactions between amide groups and other π systems, including other amides and aromatic rings, which are well documented [27]. We also added interactions between methionine sulphur atoms and aromatic rings that have been shown to be recurrent in protein structures [28], [29], [30].

#### Arpeggio web server implementation

The web implementation of Arpeggio allows the user to upload a structure or to select a file from the PDB (Fig. 1). The user can calculate interactions for a particular heteroatom group and its binding site, such as for a small-molecule ligand or interactions between chains (i.e., protein–protein and protein–DNA interactions). The user can also make a custom selection for calculation of binding-site interactions using a simple selection syntax. On completion, the user is presented with a summary of the interactions made by the entity of interest (Fig. 2a), interactive, and WebGL-based visualisation of the calculated interatomic interactions (Fig. 2b).

Additionally, the user can download a PyMOL session file containing the submitted protein structure and visualisations of the calculated interactions (Fig. 3), and tab-separated output files enumerating the calculated atom–atom contacts and aromatic ring interactions. Different types of interactions and distances are represented by connecting lines of various colours and styles, which can be enabled or disabled at the user's convenience. Examples of the representation are shown in Fig. 3.

### Discussion and Conclusions

Knowledge of the specific interactions made in macromolecular binding sites can provide insights into understanding molecular recognition, for example, in target–ligand interactions in drug development. We present Arpeggio, a freely available tool to calculate, visualise, and understand these interactions. We have found that analysis of interactions using Arpeggio, a powerful tool, has shed light on the role of mutations in genetic diseases and drug resistance [31], [32], [33], [34], [35], [36], [37], [38]. Arpeggio joins a range of publicly available software for understanding interatomic interactions. In developing Arpeggio, we have improved on atom typing and interatomic contact definitions of previous methods and have added more known interatomic interaction types than have been previously supported. Arpeggio builds on published methods for calculating interatomic interactions because of the wide range of contact types that it offers, and it stands out in having the ability to calculate interactions between any molecular entities of interest, including protein–protein and protein–nucleic acid interactions, and in visualising these both in a web interface through a state-of-the-art WebGL-based protein structure viewer and as a downloadable, stand-alone PyMOL session.

## Acknowledgements

First and foremost, we thank Adrian Schreyer; without his pioneering work on CREDO, Arpeggio would not have been possible. We are grateful to Douglas Pires for helpful discussions. H.C.J. was supported by the Biotechnology and Biological Sciences Research Council and UCB [BB/J500574/1]. B.O.-M. was supported by the Bill and Melinda Gates Foundation. D.B.A. is the recipient of a C. J. Martin Research Fellowship from the National Health and Medical Research Council of Australia (APP1072476) and is funded by the Jack Brockhoff Foundation (JBF 4186, 2016) and a Wellcome Trust Programme Grant to TLB (093167/Z/10/Z). D.B.A. and T.L.B. are funded by a Newton Fund RCUK-CONFAP Grant awarded by The Medical Research Council and Fundação de Amparo à Pesquisa do Estado de Minas Gerais (MR/M026302/1). T.L.B. wishes to acknowledge the University of Cambridge and The Wellcome Trust for facilities and support. This work builds on work funded by the Wellcome Trust.

**Competing financial interests**: The authors have no competing financial interests to declare.

## Figures and Tables

**Fig. 1:**
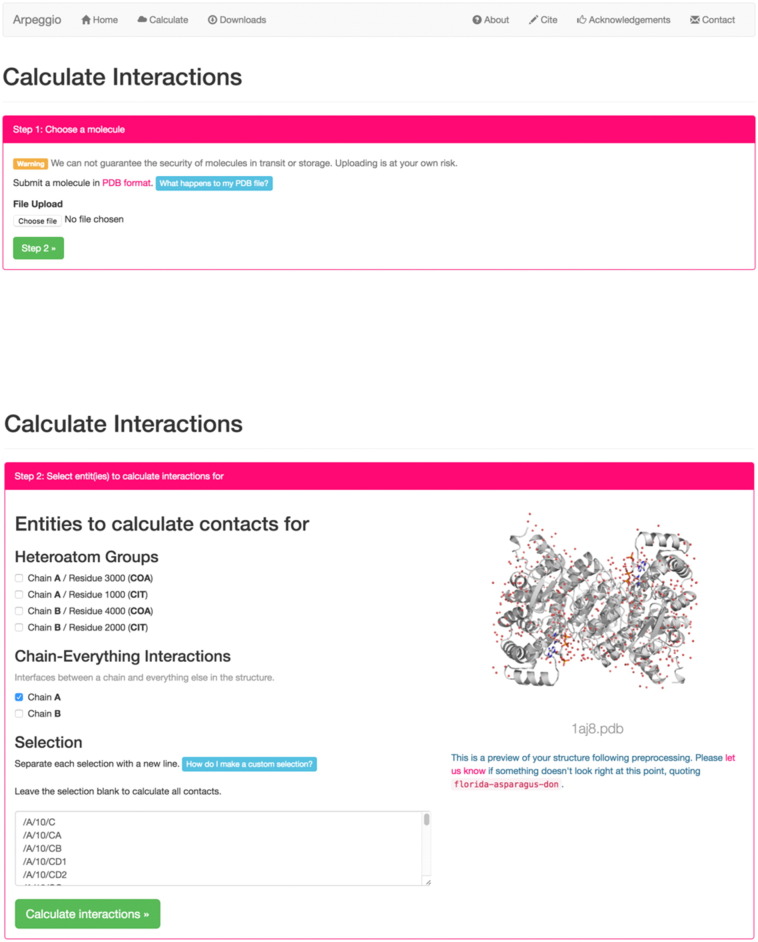
Structure submission (top) and calculation selection (bottom) pages for the Arpeggio web server. Users are prompted to select a PDB format file to submit for interaction calculations. Further information on the PDB processing step is provided in hover-over buttons (light blue) that display a pop-up dialogue box. Following structure file submission, users are presented with a preview of their structure generated by PyMOL (The PyMOL Molecular Graphics System, Version 1.8 Schrödinger, LLC) and a list of molecular entities to select from for which inter-entity interactions can be calculated. BioPython is used to detect molecular entities during the structure submission step. Users are able to enter custom entity selections at chain, residue, and atom resolution.

**Fig. 2:**
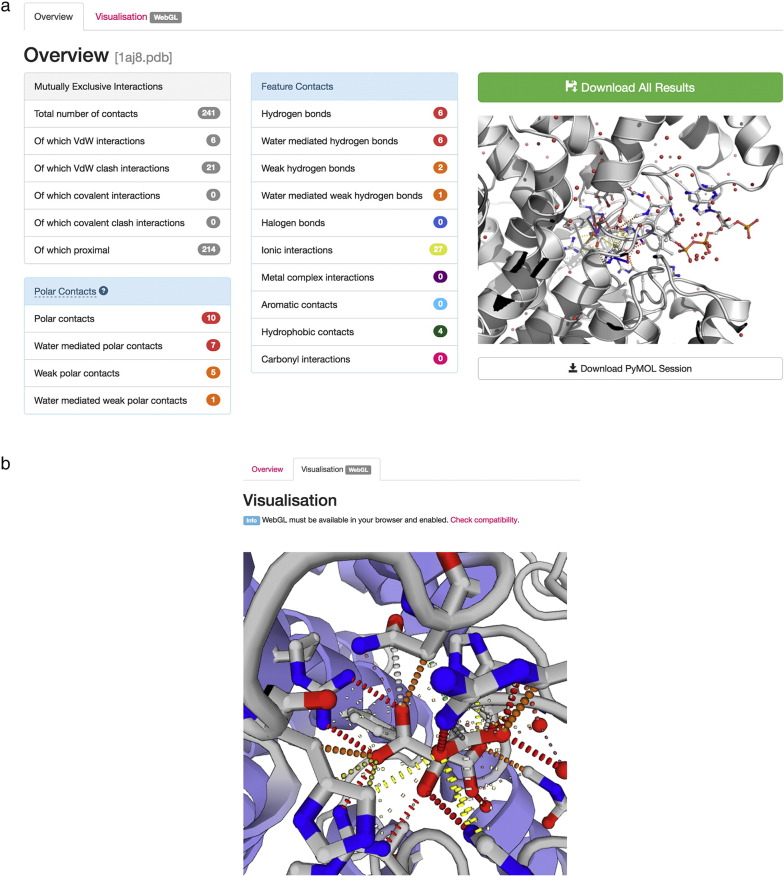
(a) Results page for an Arpeggio web server job. The pages give a summary of the interatomic interactions made by the user's molecular entity of interest. Sums of contacts are tabulated, and an image visualising interactions in the binding site is generated on-the-fly using PyMOL (The PyMOL Molecular Graphics System, Version 1.8 Schrödinger, LLC). Large buttons allow the user to download a PyMOL session file containing the structure and interactions, and tabulated results files including the results from atom-typing and interaction detection. (b) Interactive 3D, WebGL-based visualisation of interactions within the Arpeggio web interface. WebGL-based visualisation is accessible by clicking on the “Visualisation” tab on the calculation's results page. Dashed bonds are used to visualise Arpeggio-calculated non-bonded interatomic interactions. PV Viewer (http://dx.doi.org/10.5281/zenodo.20980), a WebGL protein structure viewer (https://github.com/biasmv/pv), is used for structure visualisation using custom geometries to visualise interatomic contacts. Binding site interactions between citrate and citrate synthase are shown as an example (PDB: 1AJ8) in both panels (a and b).

**Fig. 3:**
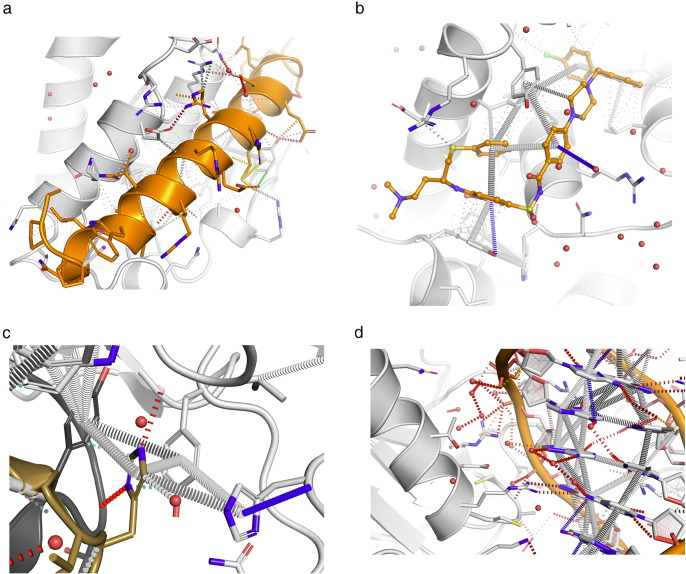
Illustrations of Arpeggio interatomic interaction visualisations. (a) BCL-XL in complex with BAD peptide (b) BCL-XL in complex with ABT-737 (c) Epidermal growth factor receptor extracellular domain in complex with inhibitory antibody GC1118A (d) p53 core DNA-binding domain in complex with DNA. Different types of non-covalent interactions are illustrated by different, coloured dashed bonds; for example, blue for halogen bonding interactions, green for hydrophobic interactions, and red for hydrogen bonding interactions. The thickness of each dash denotes the distance of the interaction; the thickest dashes indicate overlapping van der Waals' radii, while the thinnest show interactions that are “proximal”, that is, beyond van der Waals' radius overlap but within 5 Å.

## Appendix A Supplementary Data

Supplementary material Image 2
