# Supplementary material for: Arpeggio: A Web Server for Calculating and Visualising Interatomic Interactions in Protein Structures
Source: J Mol Biol. 2017 Feb 3;429(3):365–71. doi: 10.1016/j.jmb.2016.12.004 (PMC5282402; doi:10.1016/j.jmb.2016.12.004)
Supplement: Supplementary file 1 — Supplementary material [file mmc1.docx]

**Supplementary Material**

Arpeggio: a web server for calculating and visualising interatomic interactions in protein structures.

Harry C Jubb^a,^*^,#^; Alicia P Higueruelo^a,#^; Bernardo Ochoa-Montaño^a^; Will R Pitt^b^; David B Ascher^a,^*; Tom L Blundell^a,^*

^a^Department of Biochemistry, Sanger Building, University of Cambridge, 80 Tennis Court Road, Cambridge, CB2 1GA, UK.

^b^UCB , 208 Bath Road, Slough, West Berkshire SL1 3WE, United Kingdom.

^#^Present Address:

H.C.J. Wellcome Trust Sanger Institute, Wellcome Trust Genome Campus, Hinxton, Cambridge, UK;

A.P.H. Cambridge Crystallographic Data Centre , 12 Union Road, Cambridge, CB2 1EZ, United Kingdom.

* Corresponding author: T.L.B. at tlb20@cam.ac.uk, Tel: +44 1223 333628. Correspondence may also be addressed to D.B.A at [dascher@svi.edu.au](mailto:dascher@svi.edu.au) or H.C.J at [hj4@sanger.ac.uk](mailto:hj4@sanger.ac.uk).

**Supplementary Text**

## Descriptions of Interaction Types

Covalent Bonds

Covalent bonding between two atoms describes the sharing of valence electrons between the two atoms. These interactions are relatively strong.

Atoms that are covalently bonded are usually defined as being in the same molecule, and thus are not usually part of intermolecular interaction. However, there are some cases in which two initially separate molecules may conditionally interact by covalent bonds. For example, “suicide” enzyme inhibitors such as aspirin and penicillin permanently inhibit their target enzymes via covalent attachment resulting in the disruption of the enzyme’s catalytic mechanism. Intermolecular covalent attachments have also been used as chemical tools, for example in the covalent tethering of chemical fragments to protein surfaces in order to reduce the entropic penalties associated with diffusion-searching for a binding site in the development of PPI inhibitors from small-molecule fragments (Erlanson et al., 2004; Lodge et al., 2014). Thus, while Arpeggio was not intended to study intramolecular covalent interactions, we added covalent bonding definitions to understand cases of covalent bonding that are essentially intermolecular.

We also implemented a “covalent clash” definition in Arpeggio, applied when the distance between two atom centroids is less than the sum of their covalent radii. The covalent-clash definition highlights steric clashes in molecules, which may result from modelling errors in X-ray crystal structures.

van der Waals (VDW) Interactions

The spherical space around an atom’s nucleus in which electrons may be present constitutes that atom’s VDW radius. An atom’s VDW radius approximates the three dimensional space inhabited by an atom as a sphere.

The distribution of electrons in an atom’s VDW sphere may not be even. Electronegative atoms covalently bound to more electropositive atoms may be relatively more electron rich, and other electronic effects such as withdrawal of electrons into aromatic rings may also affect electron distribution throughout a molecule. Existence of dipoles in molecules, whether permanent as in the latter cases, or temporary as in instantaneous dipole induction can induce non- covalently bound molecules to interact. These interactions are known as VDW interactions (Margenau, 1939).

VDW interactions constitute three types of interatomic forces. Firstly, interactions where two permanent dipole moments interact through a pseudo charge-charge interaction are known as Keesom forces (Margenau, 1939). Secondly, permanent atomic dipoles may induce transient, instantaneous dipoles in the electron distribution of nearby neutral atoms’ VDW radii; these permanent-instantaneous dipole interactions are known as Debye forces (Margenau, 1939). Finally, London dispersion forces refer to the interactions of two instantaneous atomic dipoles, that occur stochastically as electron distributions within each atom’s VDW shell fluctuate at random or through constantly varying attraction and repulsion to the nearby electron density of other atoms (Margenau, 1939).

Individual VDW contributions are weak on their own however many VDW interactions in combination can provide much increased intermolecular affinity; the forces are taken to be additive (London, 1937; Hamaker, 1937).

While the Lennard-Jones potential (Jones, 1924) provides a mathematical description of the strength of attraction or repulsion of individual VDW interactions between two atoms, VDW interactions can be approximated in static molecular models by assessing distances between atom pairs.

In Arpeggio, for computational speed and simplicity, pairwise atomic interactions are labelled as being VDW providing that the atoms are at a distance from each other equal to the sum of their VDW radii plus an error margin of 0.1 ̊A, following Schreyer and Blundell (2009) and Schreyer and Blundell (2013). The VDW radii used were derived from tabled values in OpenBabel, rather than by looking at the electron density of the molecular structure, the data for which are not always readily available.

Hydrogen Bonds

Hydrogen-bonding refers to directional, electrostatic-esque interactions between a lone pair of electrons of an electronegative atom (typically oxygen, nitrogen or fluorine) and a hydrogen atom attached to another electronegative atom. Hydrogen-bonding has previously been considered a Keesom dipole-dipole type interaction, however analysis of bond lengths, bond stretching and spectral shifts indicate that hydrogen bonds consist of multiple components that may be difficult to de-convolute (Arunan et al., 2011), including covalent and electrostatic character in addition to dipole effects (Emsley, 1980). The multicomponent nature of hydrogen-bonding results in interactions that are stronger than VDW forces and that range in strength dependent heavily on the distance, angle between, and dielectric environment of the hydrogen bonded atoms, thus the motivation for their categorisation as separate from other weak intermolecular forces. The unique properties of hydrogen bonds as being stronger than other weak intermolecular interactions make them responsible for a number of interesting physical and biological phenomenon, including the relatively high boiling point of water, the specificity of DNA base pairing, and specific folding interactions in proteins, for example α-helix folding.

Hydrogen bonding between atom pairs requires a hydrogen bond donor and acceptor. Hydrogen bond donors contribute the hydrogen constituent of the interaction; acceptors are the non-bonding electron pair component.

Hydrogen-bonding-type interactions are not limited to using single atom oxygen, nitrogen or fluorine acceptors. For example, delocalised π orbitals of aromatic rings are electron rich and can act as non-canonical hydrogen bond acceptors.

Programs for Hydrogen Bond Detection

A number of programs exist specifically for the detection of hydrogen-bonding interactions in molecular structures. HBPLUS (McDonald and Thornton, 1994) is an early example that can effectively determine hydrogen-bonding in protein-only structures by taking careful consideration of hydrogen placement for specific amino acids. However, HBPLUS is less effective when considering non-protein interactions such as PLI interactions. The program JOY comes with a program HBOND for calculating hydrogen-bonding interactions (Mizuguchi et al., 1998). By default HBOND uses simple distance cutoffs between polar atoms to define hydrogen-bonding, however the program does have the capability to use angle criteria based on addition of hydrogen atoms to the structure. The molecular dynamics program GROMACS contains a program in its suite for detecting hydrogen- bonding based on simple atom typing and angle criteria (Lindahl et al., 2001; Van Der Spoel et al., 2005; Pronk et al., 2013). The GROMACS hydrogen-bonding detection program is designed for tracking hydrogen-bonding formation over time in molecular dynamics simulations. Given that the use of forcefields should optimise the positioning of hydrogen atoms, analysis of hydrogen-bonding over a molecular dynamics trajectory in this way should lead to higher accuracy of hydrogen-bonding determination. However, such analysis is unfeasible on a large-scale. The macromolecular visualisation program PyMOL (Schrödinger, 2015) also has facilities for analysing polar contacts, including a crude implementation of hydrogen bond detection. All of these programs suffer from limitations with respect to defining hydrogen-bonding interactions from static molecular models. Additionally, due to differences in implementation, there may be disagreements in hydrogen bond placements between different programs’ output.

While existing programs can define hydrogen bonding, and HBPLUS was used to define hydrogen bonding in the existing databases in our laboratory, they are not without their limitations, and their addition to Arpeggio would result in additional dependency and licensing issues. Therefore, we implemented hydrogen bond detection within Arpeggio.

Hydrogen Bond Detection In Arpeggio

Hydrogen bonding in Arpeggio was defined following CREDO, using SMARTS (Bone et al., 1999) based atom typing in conjunction with distance and angle definitions. Hydrogen-bonding is defined as being fulfilled if the criteria in the following are met:

distance(donor H, acceptor) ≤ rdonor H + racceptor + 0.1 ̊A & angle(donor, donor H, acceptor) ≥ 90◦

Where r refers to VDW radii. We used OpenBabel to add hydrogen positions in Arpeggio if they were not resolved in the input structure.

Difficulties in Defining Hydrogen-Bonding

The static nature of molecular structures presents several challenges for detection of hydrogen bonds. While hydrogen-bonding formation and strength is well known to be highly dependent on distance and directionality (Legon and Millen, 1987), which can be easily measured in 3D geometry, measurement of interaction angle is sensitive to hydrogen placement, in addition to resolution and dynamics-sensitive placement of heavy atoms. Macromolecular structures solved by X-ray crystallography are typically not of high enough resolution to resolve hydrogen atoms. Therefore in order to calculate angle terms in hydrogen bond detection, hydrogen atoms must be added to most structures computationally. Cheminformatics toolkits such as OpenBabel and OpenEye’s OEChem are capable of hydrogen addition for macromolecular structures. However by default, hydrogen placement is through generic idealised geometries and is not optimised. OEChem has a function, OESet3DHydrogenGeom, which uses heuristics that take into account experimentally determined hydrogen bond lengths and the location of surrounding acceptor atoms to rapidly improve hydrogen placement. OESet3DHydrogenGeom was used in the development of CREDO with OpenEye, however no equivalent function exists in the open source Open Babel toolkits. In Arpeggio we implemented hydrogen geometry optimisation using Open Babel’s built in implementations of the Merck Molecular Forcefield 94 (MMFF94) (Halgren, 1996) and Universal Forcefield (UFF) (Rapp ́e et al., 1992) forcefields, fixing non-hydrogen atoms in place and allowing hydrogens to be moved based on favourability of their interactions as determined by simulation. However, for macromolecular structures the optimisation process is slow, and it was difficult to assess the success of the process.

In addition to hydrogen placement ambiguities, the possibility of alternate protonation and tautomeric states precludes optimal detection of hydrogen-bonding interactions. Protonation states are dependent on the solvent pH and acid dissociation constant (pKa) of ionisable groups, which are dependent on the local structural environment in which each group residues. pKa values can be predicted for protein and PLI structures on a small scale (Olsson et al., 2011; Søndergaard et al., 2011). Tautomeric forms are more difficult to predict, because they are likely to be present in equilibria and are highly sensitive to pH, temperature and the nature of the solvent and local environment they are dissolved in (Antonov, 2013). Recently, Bietz et al. published Protoss, a program for adding hydrogens to PLI complexes that, following initial hydrogen placement, enumerates protonation and tautomeric states and assigns states and hydrogen geometries in such a way that a global structural hydrogen-bonding network is optimised (Bietz et al., 2014). Protoss arguably creates the most reasonable hydrogen atom placements possible for static protein and PLI complex structures. However, the program is only widely available as a web server, and while pre-calculated data are available for the wwPDB, these data are of the wwPDB’s asymmetric unit repository as opposed to using biological assemblies. Currently, the Arpeggio web server does not use hydrogens from pre-protonated structures, however the Arpeggio standalone program can do this with a command-line option.

Even with an optimal hydrogen placement algorithm for static macromolecular structures available, hydrogen bond determination is further confounded by the fact that molecules are dynamic. Dynamics presents two specific problems for hydrogen bond determination. Firstly, changes in conformation or rotameric states may mean that a hydrogen bond donor or acceptor may be able to access more than one cognate acceptor or donor. Secondly, even if a hydrogen bond donors’ heavy atom placement is relatively static, it is possible for multiple potential acceptor atoms to be within hydrogen-bonding radius. In addition to creating hydrogen placement complications as described above, it is non-trivial to determine the equilibrium at which a given hydrogen bond donor will be hydrogen bonded to one or more alternative hydrogen bond acceptors. For both problems, there is a risk of over or under assessing the number of hydrogen bonds made. Furthermore, issues in reliably assigning bond orders to small molecules, especially poorly modelled small molecule ligands, can cause issues in correct hydrogen placement.

Without an optimal solution for the hydrogen placement problem, we resorted to using the default hydrogen placements provided by Open Babel. Additionally, a simple compromise methodology for the detection of potential hydrogen-bonding interactions that is not sensitive to the hydrogen placements that we utilised in Arpeggio, is described below. While such a method might over-represent hydrogen-bonding interactions, it is a reasonable compromise that has been utilised in previous analyses.

Weak Hydrogen Bonds

Weak hydrogen-bonding-type interactions may also be made between hydrogen bond acceptors and hydrogens bonded to heavy atoms that are only weakly electronegative, i.e. weak donors (Desiraju and Steiner, 1999). These interactions are typically longer in distance than conventional hydrogen bonds, tending to be within VDW distances rather than shorter than VDW distances.

Following CREDO, we utilised a SMARTS based definition of weak hydrogen bond donors, including all non-quaternary carbon atoms (see section below). Distance and angle terms follow those of hydrogen-bonding.

“Polar” Contacts

Limitations in protein structure data and the PDB file format cause difficulties elucidating hydrogen bonds precisely for protein structures.

Given the dependence of an atom type, distance, and angle-based definition of hydrogen-bonding on “correct” hydrogen placement, We implemented less restrictive hydrogen-bonding- like contact definitions in Arpeggio. “Polar” contacts use hydrogen-bonding definitions with stricter distance criteria but no angle criteria. These looser definitions of contacts between hydrogen bond donors/acceptors address the lack of accountability by hydrogen placement-sensitive definitions for molecular dynamics or potential resonance forms when analysing static crystal structures of proteins. The angle-less definition of hydrogen-bonding is reminiscent of that used in the program HBOND (Mizuguchi et al., 1998). We also added a “weak polar” contact definition covering angle independent weak hydrogen-bonding type interactions.

Halogen Bonds

Hydrogen-bonding is not the only kind of non-ionic, electrostatic-type non-covalent interaction. Halogen atoms are anisotropically polarisable, allowing them to make directionally-dependent head-on “charge-transfer” interactions with electron-rich (δ−) hydrogen bond acceptors excluding fluorine (Metrangolo and Resnati, 2001; Auffinger et al., 2004). Like hydrogen bonds, halogen bond distances can be shorter than the sum of the donor and acceptor VDW radii, indicative of the electrostatic nature of this non-covalent interaction.

Halogen bonding is particularly important in drug discovery, as halogens can be incorporated into synthetic molecules to enhance interactions with target protein mainchain carbonyls (Pollock et al., 2015) and amide π systems, and sidechain carboxylates and ring π systems. However, in its current iteration Arpeggio is limited to atom-atom halogen bonding contacts.

Ionic Interactions

Ionic interactions refer to strong electrostatic bonding between two ions carrying opposing formal charges. Electrostatics are important drivers in protein:small-molecule and protein- protein binding (Wade, 1998; Sheinerman et al., 2000).

Calculation of accurate ionisation potentials in macromolecular structures is difficult without computationally expensive pKa calculations. While these calculations are possible in programs such as PROPKA (Søndergaard et al., 2011; Olsson et al., 2011), licensing restrictions and a desire to keep the program runtime down to a suitable level restrict their use as an addition to an open source tool such as Arpeggio.

In Arpeggio, we used SMARTS queries to account for the ambiguous state of some chemical groups as being potentially positively charged or potentially negatively charged. We implemented Arpeggio’s SMARTS-based atom typing to label applicable groups as either positively ionisable or negatively ionisable, following Schreyer and Blundell (2013).

Metal Complexes

Metal complexes refer to the coordination of atoms or groups of atoms to a metal ion. As metal ions carry positive formal charges, lone pairs of electrons, such as hydrogen bond acceptor lone pairs, can form dative covalent bonds to empty metal molecular orbitals. Metal complexes take on a range of roles in protein structure, including structural/fold assistive roles such as in zinc finger motifs (Pavletich and Pabo, 1991), and catalytic roles in enzyme active sites (Andreini et al., 2008).

While a “metal complex” may refer to a metal bound to all its ligands, in Arpeggio we defined metal complex interactions on a pairwise, atom-to-atom basis. Metal complex contact criteria were on the basis of a 2.8 Å distance between a metal atom or ion (as perceived by OpenBabel) and a hydrogen bond acceptor as detected by SMARTS.

Aromatic Ring Interactions

Aromatic ring structures differ from saturated cyclic moieties in that they have delocalised π electron systems above and below the plane of the ring. Electron-rich ring faces can possess a partial negative charge and can be analogous to hydrogen bond acceptors, while electron deficient ring edges can possess a partial positive charge. These properties of aromatic rings enable their involvement not only in Keesom and Debye type VDW forces but also pseudo-hydrogen-bonding type interactions. Thus, aromatic rings can make biologically meaningful interactions with other rings, atoms and groups. Chourasia et al. found that the majority of proteins in the wwPDB have π interaction networks (Chourasia et al., 2011).

Ring interactions are prevalent in biological macromolecules. For example, DNA base- stacking is in part a product of ring-ring π orbital overlaps (Martinez and Iverson, 2012). Aromatic interactions have been found to be important in peptide folding (Mahalakshmi et al., 2005; Bakota et al., 2013; Mahalakshmi et al., 2006; Tatko and Waters, 2002; Kar et al., 2009; Sengupta et al., 2005; Aravinda et al., 2003) and in globular protein folding (Burley and Petsko, 1985; Thomas et al., 2002; Hunter et al., 1991; McGaughey et al., 1998; Bhattacharyya et al., 2002; Pashuck and Stupp, 2010), where two or more multiple aromatic moieties can form stacks (Lanzarotti et al., 2011). Aromatic interactions have been exploited in rational drug design (Babine and Bender, 1997; Silva et al., 2006) and even used in the making of molecular tweezers (Sygula et al., 2007) and nanotubes (Crisma et al., 2006).

Chakrabarti and Bhattacharyya (2007) categorised ring interaction geometries into nine classes based on the angles of one ring’s plane and normal to another. Simulation studies suggest that ring interaction geometries can be distance dependent (Chelli et al., 2002), although these simulations were conducted in vacuo, water and organic solvents rather than protein environments. We used the Chakrabarti and Bhattacharyya (2007) categories to assess ring-ring interactions in Arpeggio, following CREDO (Schreyer and Blundell, 2013). These ring-ring interactions are separate from atom-based aromatic-aromatic atom interactions, which are also calculated in Arpeggio.

Atom-Ring Interactions

Electron rich π orbitals above and below the planes of aromatic rings can also interact with a variety of partially or formally positively charged atoms. These include cations (Ma and Dougherty, 1997; Biot et al., 2003; Crowley and Golovin, 2005), hydrogen bond donors (Tewari and Dubey, 2008), halogen bond donors (Auffinger et al., 2004), and weakly electropositive carbon atoms (Tewari and Dubey, 2008). Methionine sulphur atoms have also been shown to interact with π density in proteins (Zhou et al., 2009a; Valley et al., 2012; Biswal et al., 2012). With the exception of Met-S:π interactions, which we added to Arpeggio, these interactions were defined as in CREDO (Schreyer and Blundell, 2009; Schreyer and Blundell, 2013).

Groups of Atoms’ Interactions

Groups of atoms can take on electronic properties as a whole, such as amide groups. While generic detection of interactions between groups of atoms and other groups of atoms or aromatic rings would be ideal, technological limitations make generic solutions challenging to implement. Thus, in Arpeggio we implemented a limited array of important group-ring and group-group interactions, specifically those involving amide groups, using manual definitions of atom groups by SMARTS.

Group-Ring Interactions

The premise of partially charged atoms interacting with localised positive and negative charges surrounding aromatic rings can be extended to ring interactions with groups of atoms. For example, interactions of aromatic rings with amide groups were identified in the early years of structural biology (Burley and Petsko, 1986) and noted as electrostatic-type interactions.

These group interactions are due to amide bonds having π electron density above and below the plane of the amide bond, analogous to the π systems above and below the planes of electron delocalised aromatic rings. We added distance and angle based criteria to detect amide-π interactions, using the geometric centre of the amide bond as the reference point for interactions originating from amide SMARTS defined in the section below.

Group-Group Interactions

Amide π systems also enable amides to stack in the same manner as aromatic rings, above and below the plane of the amide group atoms (Imai et al., 2009). These interactions may be especially important in protein folding (James et al., 2009). We used distance and angle based criteria to detect these interactions between amide groups (see Appendix A.2).

Hydrophobic Interactions

Hydrophobic interactions refer not to direct, enthalpically favourable interactions between hydrophobic groups, but to aggregation of hydrophobic moieties due to a number of thermodynamic effects. Hydrophobic aggregation minimises entropy loss from water maximising hydrogen-bonding network potential by forming rigid clathrate shells around hydrophobic solutes (Hartley, 1936; Haselmeier et al., 1995; Nakahara et al., 1996; Silverstein, 1998). Thus hydrophobic aggregation maximises entropy gains by ejecting a maximal proportion of water molecules into bulk solvent, and balances the enthalpic penalty of breaking dynamic hydrogen bonds (and entropic penalty of solvation shell rigidification) with enthalpic gains from maximally satisfied, rigid (and therefore strong) hydrogen-bonding in solvation shells (Silverstein, 1998; Herzfeld and Olbris, 2002).

Because of the indirect nature of the hydrophobic effect and the complex thermodynamic balances involved, measurement and quantification of hydrophobic “interactions” is non- trivial. Thus any assessment of the hydrophobic effect purely from molecular structure must be taken with a pinch of salt. Nonetheless, hydrophobic interactions can be assessed in molecular structures on a whole molecule interaction level using apolar buried surface area, calculated using the programs like NACCESS (Hubbard and Thornton, 1996), or OpenEye (OpenEye Scientific Software, 2010). Atomic contributions to apolar surface area can also be assessed using these methods. With the ability of existing methods to assess apolar interactions in mind, we elected to implement hydrophobic interactions in Arpeggio as pairwise interatomic interactions between atoms typed as hydrophobic (providing that they match a SMARTS query). Like the majority of interactions defined in Arpeggio, this definition follows the existing CREDO, PICCOLO and BIPA definitions (Bickerton, 2009; Bickerton et al., 2011; Lee and Blundell, 2009b; Schreyer and Blundell, 2009; Schreyer and Blundell, 2013).

Carbonyl Interactions

Carbonyl groups, such as those in protein mainchain backbones, have a Cδ+=Oδ− dipole due to the oxygen atom being comparatively more electronegative than the carbon. Therefore, carbonyl oxygen atoms can form productive, directionally dependent, non-covalent interactions with carbonyl carbon atoms from different carbonyl groups that are adjacent in 3D space. Carbonyl-carbonyl interactions are similar to and can be equated with hydrogen-bonding-type interactions, sharing similar energetic strength to hydrogen-bonding, and have been noted for their ability to stabilise otherwise unstable mainchain conformations in proteins (Deane et al., 1999). While these interactions have been traditionally considered as charge-charge type interactions, recent analysis suggest that resonance/electron delocalisation is the contributor to carbonyl-carbonyl interactions; in molecular orbital terms, these interactions equate to oxygen lone pair (n) interaction with carbon π anti-bonding orbitals (Cπ) (Choudhary et al., 2009). Therefore, following CREDO, we defined carbonyl-carbonyl interactions by distance but not angle criteria.

3.2.13 Mutual Exclusivity of Interactions

Interatomic interactions defined by distances and angles in macromolecular structures can be of multiple types. For example, a hydrogen bond donor/acceptor pair may also be atom-typed as positive and negative ionisable, thus an ionic interaction may be detected in addition to a hydrogen-bonding pair.

The philosophy we took in the implementation of Arpeggio towards the potential for detecting multiple, overlapping contact types between atom pairs follows that of CREDO (Schreyer and Blundell, 2013) in labelling all possible detected interactions, rather than attempting to make a decision on a contact type without basis. Thus, the “feature” contact types in Arpeggio, which include contact definitions involving atom typing, were non-mutually exclusive. However, the five distance-only based contact types (covalent clash, covalent, VDW clash, VDW, and proximal) are mutually exclusive, because an atom pair within the interaction detection radius can only fit into one distance criterion.

## Atom and Contact Typing

ATOM_TYPES = {

 "hbond acceptor":
 {
 "acceptor" : "[#8,#9,$([#16;H0,H1;v2,v1]),$([N;v3;!$(N-*=!@[O,N,P,S]);!$(N-!@a);!$([NH]=!@*)]),$([nH0;+0])]",
 "enol" : "[$([nH]:@c(=O))]",
 "tautomeric nH" : "[$([n;H1;v3;!$([nH]cccc)])]",
 # AMBIGUITY OF TERMINAL AMIDES MAY AFFECT NON-PROTEIN AMIDES
 "NH2 terminal amide": "[$([N;H2;v3;$(N-C(=O))])]"
 },

 "hbond donor":
 {
 "donor" : "[N!H0v3,N!H0+v4,OH+0,SH+0,nH+0]",
 "oxygen acid" : "[$([O;H0;$(O=C([OH])-*)])]",
 "tautomer nH" : "[$(n:a:[nH])]",
 # AMBIGUITY OF TERMINAL AMIDES MAY AFFECT NON-PROTEIN AMIDES
 "oxygen amide term" : "[$([O;H0;$(O=C-[NH2])])]"

 },

 "xbond acceptor":
 {
 # SAME AS HBA
 "acceptor" : "[#8,#9,$([#16;H0,H1;v2,v1]),$([N;v3;!$(N-*=!@[O,N,P,S]);!$(N-!@a);!$([NH]=!@*)]),$([nH0;+0])]",
 "enol" : "[$([nH]:@c(=O))]",
 "tautomeric nH" : "[$([n;H1;v3;!$([nH]cccc)])]",
 # AMBIGUITY OF TERMINAL AMIDES MAY AFFECT NON-PROTEIN AMIDES
 "NH2 terminal amide": "[$([N;H2;v3;$(N-C(=O))])]"
 },

 "xbond donor":
 {
 "donor" : "[Cl,Br,I;X1;$([Cl,Br,I]-[#6])]"
 },

 "weak hbond acceptor":
 {
 # SAME AS HBA
 "acceptor" : "[#8,#9,$([#16;H0,H1;v2,v1]),$([N;v3;!$(N-*=!@[O,N,P,S]);!$(N-!@a);!$([NH]=!@*)]),$([nH0;+0])]",
 "enol" : "[$([nH]:@c(=O))]",
 "tautomeric nH" : "[$([n;H1;v3;!$([nH]cccc)])]",
 # AMBIGUITY OF TERMINAL AMIDES MAY AFFECT NON-PROTEIN AMIDES
 "NH2 terminal amide": "[$([N;H2;v3;$(N-C(=O))])]",
 "c-x halogens" : "[Cl,Br,I;X1;$([Cl,Br,I]-[#6])]"
 },

 "weak hbond donor":
 {
 "donor" : "[#6!H0]"
 },

 # SEE RDKIT `BaseFeatures.fdef`
 "pos ionisable":
 {
 "rdkit basic group" : "[$([N;H2&+0][C;!$(C=*)]),$([N;H1&+0]([C;!$(C=*)])[C;!$(C=*)]),$([N;H0&+0]([C;!$(C=*)])([C;!$(C=*)])[C;!$(C=*)]);!$(N[a])]",
 "imidazole" : "[n;R1]1[c;R1][n;R1][c;R1][c;R1]1",
 "guanidine amidine" : "NC(=N)",
 "rdkit posn" : "[#7;+;!$([N+]-[O-])]",
 "cations" : "[$([*+1,*+2,*+3]);!$([N+]-[O-])]",
 "metals" : "[Li,Be,Na,Mg,Al,K,Ca,Sc,Ti,V,Cr,Mn,Fe,Co,Ni,Cu,Zn,Ga,Rb,Sr,Y,Zr,Nb,Mo,Tc,Ru,Rh,Pd,Ag,Cd,In,Sn,Cs,Ba,La,Ce,Pr,Nd,Pm,Sm,Eu,Gd,Tb,Dy,Ho,Er,Tm,Yb,Lu,Hf,Ta,W,Re,Os,Ir,Pt,Au,Hg,Tl,Pb,Bi,Po,Fr,Ra,Ac,Th,Pa,U,Np,Pu,Am,Cm,Bk,Cf]"
 },

 "neg ionisable":
 {
 "O acidic group" : "[$([OH,O-]-[C,S,N,P,Cl,Br,I]=O),$(O=[C,S,N,P,Cl,Br,I]-[OH,O-])]",
 "anions" : "[*-1,*-2]"
 },

 "hydrophobe":
 {
 "hydrophobe" : "[#6+0!$(*~[#7,#8,F]),SH0+0v2,s+0,Cl+0,Br+0,I+0]"
 },

 "carbonyl oxygen":
 {
 "oxygen" : "[$([OH0]=[CX3,c]);!$([OH0]=[CX3,c]-[OH,O-])]"
 },

 "carbonyl carbon":
 {
 "carbon" : "[$([CX3,c]=[OH0]);!$([CX3,c](=[OH0])-[OH,O-])]"
 },

 "aromatic":
 {
 "arom_4" : "[a;r4,!R1&r3]1:[a;r4,!R1&r3]:[a;r4,!R1&r3]:[a;r4,!R1&r3]:1",
 "arom_5" : "[a;r5,!R1&r4,!R1&r3]1:[a;r5,!R1&r4,!R1&r3]:[a;r5,!R1&r4,!R1&r3]:[a;r5,!R1&r4,!R1&r3]:[a;r5,!R1&r4,!R1&r3]:1",
 "arom_6" : "[a;r6,!R1&r5,!R1&r4,!R1&r3]1:[a;r6,!R1&r5,!R1&r4,!R1&r3]:[a;r6,!R1&r5,!R1&r4,!R1&r3]:[a;r6,!R1&r5,!R1&r4,!R1&r3]:[a;r6,!R1&r5,!R1&r4,!R1&r3]:[a;r6,!R1&r5,!R1&r4,!R1&r3]:1",
 "arom_7" : "[a;r7,!R1&r6,!R1&r5,!R1&r4,!R1&r3]1:[a;r7,!R1&r6,!R1&r5,!R1&r4,!R1&r3]:[a;r7,!R1&r6,!R1&r5,!R1&r4,!R1&r3]:[a;r7,!R1&r6,!R1&r5,!R1&r4,!R1&r3]:[a;r7,!R1&r6,!R1&r5,!R1&r4,!R1&r3]:[a;r7,!R1&r6,!R1&r5,!R1&r4,!R1&r3]:[a;r7,!R1&r6,!R1&r5,!R1&r4,!R1&r3]:1",
 "arom_8" : "[a;r8,!R1&r7,!R1&r6,!R1&r5,!R1&r4,!R1&r3]1:[a;r8,!R1&r7,!R1&r6,!R1&r5,!R1&r4,!R1&r3]:[a;r8,!R1&r7,!R1&r6,!R1&r5,!R1&r4,!R1&r3]:[a;r8,!R1&r7,!R1&r6,!R1&r5,!R1&r4,!R1&r3]:[a;r8,!R1&r7,!R1&r6,!R1&r5,!R1&r4,!R1&r3]:[a;r8,!R1&r7,!R1&r6,!R1&r5,!R1&r4,!R1&r3]:[a;r8,!R1&r7,!R1&r6,!R1&r5,!R1&r4,!R1&r3]:[a;r8,!R1&r7,!R1&r6,!R1&r5,!R1&r4,!R1&r3]:1"
 }
}

PROT_ATOM_TYPES = {
 "hbond acceptor": [
 "ALAO", # all the carbonyl Oxygens in the main chain
 "ARGO","ASNO","ASPO","CYSO","GLNO","GLUO","GLYO","HISO","ILEO","LEUO","LYSO","METO","PHEO","PROO","SERO","THRO","TRPO","TYRO","VALO",
 "ALAOXT", # all the carbonyl Oxygens terminals
 "ARGOXT","ASNOXT","ASPOXT","CYSOXT","GLNOXT","GLUOXT","GLYOXT","HISOXT","ILEOXT","LEUOXT","LYSOXT","METOXT","PHEOXT","PROOXT","SEROXT","THROXT","TRPOXT","TYROXT","VALOXT","ASNOD1",
 "ASNND2", #for the ambiguity of the position of the N and O
 "ASPOD1","ASPOD2","GLNOE1",
 "GLNNE2", #for the ambiguity of the position of the N and O
 "GLUOE1","GLUOE2",
 "HISND1", #for the ambiguity of the position of the N/C
 "HISCE1", #for the ambiguity of the position of the N/C
 "HISNE2", #for the ambiguity of the position of the N/C
 "HISCD2", #for the ambiguity of the position of the N/C
 "METSD", #http://pubs.acs.org/doi/abs/10.1021/jz300207k and pubid 19089987
 "CYSSG", #pubid 19089987, also when they from di-sulfide (Cys-Cys, fig 8 paper)
 "SEROG","THROG1","TYROH"
 ]
 ,
 "hbond donor": [
 "ALAN", # all the amide nitrogens in the main chain except proline
 "ARGN","ASNN","ASPN","CYSN","GLNN","GLUN","GLYN","HISN","ILEN","LEUN","LYSN","METN","PHEN","SERN","THRN","TRPN","TYRN","VALN","ARGNE","ARGNH1","ARGNH2","ASNND2",
 "ASNOD1", #for the ambiguity of the position of the N and O
 "CYSSG", #http://www.ncbi.nlm.nih.gov/pubmed/19089987
 "GLNNE2",
 "GLNOE1", #for the ambiguity of the position of N/O
 "HISND1", #for the ambiguity of the position of the N/C
 "HISCE1", #for the ambiguity of the position of the N/C
 "HISNE2", #for the ambiguity of the position of the N/C
 "HISCD2", #for the ambiguity of the position of the N/C
 "LYSNZ","SEROG", "THROG1", "TRPNE1","TYROH"
 ]

 ,
 "xbond acceptor": [
 "ALAO", # all the carbonyl Oxygens in the main chain
 "ARGO","ASNO","ASPO","CYSO","GLNO","GLUO","GLYO","HISO","ILEO","LEUO","LYSO","METO","PHEO","PROO","SERO","THRO","TRPO","TYRO","VALO",
 "ALAOXT", # all the carbonyl Oxygens terminals
 "ARGOXT","ASNOXT","ASPOXT","CYSOXT","GLNOXT","GLUOXT","GLYOXT","HISOXT","ILEOXT","LEUOXT","LYSOXT","METOXT","PHEOXT","PROOXT","SEROXT","THROXT","TRPOXT","TYROXT","VALOXT","ASNOD1",
 "ASNND2", #for the ambiguity of the position of the N and O
 "ASPOD1","ASPOD2","GLNOE1"
 "GLNNE2", #for the ambiguity of the position of the N and O
 "GLUOE1","GLUOE2",
 "HISND1", #for the ambiguity of the position of the N/C
 "HISCE1", #for the ambiguity of the position of the N/C
 "HISNE2", #for the ambiguity of the position of the N/C
 "HISCD2", #for the ambiguity of the position of the N/C
 "METSD", #http://pubs.acs.org/doi/abs/10.1021/jz300207k and pubid 19089987
 "CYSSG", #pubid 19089987, also when they from di-sulfide (Cys-Cys, fig 8 paper)
 "SEROG","THROG1","TYROH"
 ]
 ,
 "weak hbond acceptor": [
 "ALAO", # all the carbonyl Oxygens in the main chain
 "ARGO","ASNO","ASPO","CYSO","GLNO","GLUO","GLYO","HISO","ILEO","LEUO","LYSO","METO","PHEO","PROO","SERO","THRO","TRPO","TYRO","VALO",
 "ALAOXT", # all the carbonyl Oxygens terminals
 "ARGOXT","ASNOXT","ASPOXT","CYSOXT","GLNOXT","GLUOXT","GLYOXT","HISOXT","ILEOXT","LEUOXT","LYSOXT","METOXT","PHEOXT","PROOXT","SEROXT","THROXT","TRPOXT","TYROXT","VALOXT","ASNOD1",
 "ASNND2", #for the ambiguity of the position of the N and O
 "ASPOD1","ASPOD2","GLNOE1"
 "GLNNE2", #for the ambiguity of the position of the N and O
 "GLUOE1","GLUOE2",
 "HISND1", #for the ambiguity of the position of the N/C
 "HISCE1", #for the ambiguity of the position of the N/C
 "HISNE2", #for the ambiguity of the position of the N/C
 "HISCD2", #for the ambiguity of the position of the N/C
 "METSD", #http://pubs.acs.org/doi/abs/10.1021/jz300207k and pubid 19089987
 "CYSSG", #pubid 19089987, also when they from di-sulfide (Cys-Cys, fig 8 paper)
 "SEROG","THROG1","TYROH"
 ]
 ,
 "weak hbond donor": [
 "ALACA", # all the c-alphas
 "ARGCA","ASNCA","ASPCA","CYSCA","GLNCA","GLUCA","GLYCA","HISCA","ILECA","LEUCA","LYSCA","METCA","PHECA","PROCA","SERCA","THRCA","TRPCA","TYRCA","VALCA",
 "ALACB", #cb and further down
 "ARGCB","ARGCG","ARGCD","ASNCB","ASPCB","CYSCB","GLNCB","GLNCG","GLUCB","GLUCG","GLNCB","HISCB","ILECB","ILECG1","ILECD1","ILECG2","LEUCB","LEUCG","LEUCD1","LEUCD2","LYSCB","LYSCG","LYSCD","LYSCE","METCB","METCG","METCE","PHECB","PHECG","PHECD1","PHECD2","PHECE1","PHECE2","PHECZ","PROCB","PROCG","PROCD","SERCB","THRCB","THRCG2","TRPCB","TRPCD1""TRPCE3","TRPCZ3","TRPCH2","TRPCZ2","TYRCB","TYRCD1","TYRCD2","TYRCE1","TYRCE2","TRYCB","VALCB","VALCG1","VALCG2"
 ]
 ,
 "pos ionisable": [
 "ARGNE","ARGCZ","ARGNH1","ARGNH2","HISCG","HISND1","HISCE1","HISNE2","HISCD2","LYSNZ"
 ]
 ,
 "neg ionisable": [
 "ASPOD1","ASPOD2","GLUOE1","GLUOE2"
 ]
 ,
 "hydrophobe": [
 "ALACB","ARGCB","ARGCG","ASNCB","ASPCB",
 "CYSCB", #sulfur in Cys has an Hydrogen, it is polarised
 "GLNCB","GLNCG","GLUCB","GLUCG","GLNCB","HISCB","ILECB","ILECG1","ILECD1","ILECG2","LEUCB","LEUCG","LEUCD1","LEUCD2","LYSCB","LYSCG","LYSCD","METCB","METCG","METSD","METCE","PHECB","PHECG","PHECD1","PHECD2","PHECE1","PHECE2","PHECZ","PROCB","PROCG","THRCG2","TRPCB","TRPCG","TRPCD2","TRPCE3","TRPCZ3","TRPCH2","TRPCZ2","TRYCB","TYRCG","TYRCD1","TYRCD2","TYRCE1","TYRCE2","VALCB","VALCG1","VALCG2"
 ]
 ,
 "carbonyl oxygen": [
 "ALAO", # all the carbonyl Oxygens in the main chain
 "ARGO","ASNO","ASPO","CYSO","GLNO","GLUO","GLYO","HISO","ILEO","LEUO","LYSO","METO","PHEO","PROO","SERO","THRO","TRPO","TYRO","VALO"
 ]
 ,
 "carbonyl carbon": [
 "ALAC","ARGC","ASNC","ASPC","CYSC","GLNC","GLUC","GLYC","HISC","ILEC","LEUC","LYSC","METC","PHEC","PROC","SERC","THRC","TRPC","TYRC","VALC"
 ]
 ,
 "aromatic": [
 "HISCG","HISND1","HISCE1","HISNE2","HISCD2","PHECG","PHECD1","PHECD2","PHECE1","PHECE2","PHECZ","TRPCG","TRPCD1","TRPCD2","TRPNE1","TRPCE2","TRPCE3","TRPCZ2","TRPCZ3","TRPCH2","TYRCG","TYRCD1","TYRCD2","TYRCE1","TYRCE2","TYRCZ"

 ]
}

CONTACT_TYPES_DIST_MAX = 4.5

CONTACT_TYPES = {
 "hbond":
 {
 "distance": 3.9,
 "polar distance": 3.5,
 "angle rad": 1.57,
 "angle degree": 90.0
 },

 "weak hbond":
 {
 "distance": 3.6,
 "weak polar distance": 3.5,
 "angle rad": 2.27,
 "angle degree": 130.0,
 "cx angle min rad": 0.52,
 "cx angle min degree": 30.0,
 "cx angle max rad": 2.62,
 "cx angle max degree": 150.0
 },

 "aromatic":
 {
 "distance": 4.0,
 "centroid_distance": 6.0,
 "atom_aromatic_distance": 4.5,
 "met_sulphur_aromatic_distance": 6.0
 },

 "amide":
 {
 "centroid_distance": 6.0,
 "angle degree": 30.0,
 "angle rad": 0.52
 },

 "xbond":
 {
 "catmap distance": 1.85, # SAME AS BROMINE
 "angle theta 1 rad": 2.09,
 "angle theta 1 degree": 120.0,
 "angle theta 2 min rad": 1.22,
 "angle theta 2 max rad": 2.97,
 "angle theta 2 min degree": 70.0,
 "angle theta 2 max degree": 170.0
 },

 "ionic":
 {
 "distance": 4.0
 },

 "hydrophobic":
 {
 "distance": 4.5
 },

 "carbonyl":
 {
 "distance": 3.6
 },

 "metal":
 {
 "distance": 2.8
 }
}
